# Design and rationale of the QUAZAR Lower-Risk MDS (AZA-MDS-003) trial: a randomized phase 3 study of CC-486 (oral azacitidine) plus best supportive care vs placebo plus best supportive care in patients with IPSS lower-risk myelodysplastic syndromes and poor prognosis due to red blood cell transfusion–dependent anemia and thrombocytopenia

**DOI:** 10.1186/s12878-016-0049-5

**Published:** 2016-05-03

**Authors:** Guillermo Garcia-Manero, Antonio Almeida, Aristoteles Giagounidis, Uwe Platzbecker, Regina Garcia, Maria Teresa Voso, Stephen R. Larsen, David Valcarcel, Lewis R. Silverman, Barry Skikne, Valeria Santini

**Affiliations:** Department of Leukemia, The University of Texas MD Anderson Cancer Center, Houston, TX USA; Instituto Portugues de Oncologia Francisco Gentil, Lisbon, Portugal; Marien-Hospital Akademisches Lehrkrankenhaus, Dusseldorf, Germany; Medizinschen Fakultat Carl Gustav Carus der TU Dresden, Dresden, Germany; Hospital Clinico Universitario Virgen de la Victoria, Malaga, Spain; Universita Cattolica del Sacro Cuore, Rome, Italy; Royal Prince Alfred Hospital, Sydney, Australia; Hospital Vall D’Hebron, Barcelona, Spain; Mount Sinai School of Medicine, New York, NY USA; Celgene Corporation, Overland Park, KS USA; Hematology, University of Florence, AOU Careggi, Florence, Italy

**Keywords:** Myelodysplastic syndromes, MDS, IPSS lower risk, CC-486, Azacitidine, Red blood cell transfusion dependence, Anemia, Thrombocytopenia

## Abstract

**Background:**

CC-486 is an oral formulation of the epigenetic modifier azacitidine. In an expanded phase 1 trial, CC-486 demonstrated clinical and biological activity in patients with International Prognostic Scoring System (IPSS) lower-risk (low- and intermediate-1–risk) myelodysplastic syndromes (MDS) with poor prognostic features including anemia and/or thrombocytopenia who may have required red blood cell or platelet transfusions. The overall response rate was 40 %, including hematologic improvement in 28 % of patients and RBC transfusion independence sustained for 56 days in 47 % of patients with baseline transfusion dependence. Based on the results of this study, the randomized, placebo-controlled phase 3 QUAZAR Lower-Risk MDS trial (AZA-MDS-003) was initiated. The design and rationale for this trial comparing CC-486 with placebo for the treatment of patients with IPSS lower-risk MDS with poor prognostic features are described.

**Methods:**

Patients must have IPSS lower-risk MDS with red blood cell (RBC) transfusion–dependent anemia and thrombocytopenia. Eligible patients are randomized 1:1 to receive 300 mg of CC-486 or placebo once daily for the first 21 days of 28-day treatment cycles. Disease status assessments occur at the end of cycle 6 and patients may continue to receive treatment unless there is evidence of progressive disease, lack of efficacy, or unacceptable toxicity. The primary endpoint is RBC transfusion independence for ≥ 84 days, assessed according to International Working Group 2006 criteria. Secondary endpoints include overall survival, hematologic response including platelet response and erythroid response, RBC transfusion independence for ≥ 56 days, duration of RBC transfusion independence, time to RBC transfusion independence, rate of acute myeloid leukemia (AML) progression, time to AML progression, clinically significant bleeding events, safety, health-related quality of life, and healthcare resource utilization.

**Conclusions:**

This study will provide data on the efficacy and safety of CC-486 in the treatment of IPSS lower-risk MDS with poor prognosis due to the presence of both RBC transfusion–dependent anemia and thrombocytopenia. Positive results of the AZA-MDS-003 study may expand treatment options for patients with IPSS lower-risk MDS.

**Trial registration:**

ClinicalTrials.gov NCT01566695, registered March 27, 2012.

**Electronic supplementary material:**

The online version of this article (doi:10.1186/s12878-016-0049-5) contains supplementary material, which is available to authorized users.

## Background

Myelodysplastic syndromes (MDS) are a heterogeneous group of clonal myeloid malignancies characterized by ineffective hematopoiesis, peripheral cytopenias, recurring cytogenetic and molecular abnormalities, and risk of progression to acute myeloid leukemia (AML) [1–3]. More than 10,000 new cases in the United States and 8000 in Europe are estimated to occur each year [4, 5]; however, because of its heterogeneity, MDS is challenging to diagnose and classify [6]. MDS is primarily a disease of older age. People aged ≥ 65 years have an annual incidence of MDS of 75 per 100,000 persons, whereas the incidence is estimated at only 3.3 per 100,000 persons in the general population [7]. Due to the heterogeneity of outcomes associated with this disease, patients are generally stratified by risk to aid treatment decision making [8]. The International Prognostic Scoring System (IPSS) is a standard tool used for risk assessment of adult patients with de novo untreated MDS [9]. The IPSS stratifies patients into 4 risk groups: low, intermediate (Int)-1, Int-2, and high risk, based on cytogenetics, percentage of bone marrow blasts, and number of cytopenias. Patients with IPSS lower-risk (low and Int-1) MDS account for approximately two-thirds of patients and are generally viewed as having a favorable prognosis. However, a subset of patients in the IPSS lower-risk group have additional poor prognostic factors that limit survival, and these patients may benefit from early therapeutic intervention [10, 11]. More recently, the IPSS-R has been developed to further refine the IPSS [12].

Anemia and thrombocytopenia are among the most common characteristics in patients with lower-risk MDS, and their severity may impact prognosis [9, 10]. A study of 856 patients with IPSS lower-risk MDS found that baseline factors including thrombocytopenia, anemia at hemoglobin levels requiring red blood cell (RBC) transfusion, older age, higher percentage of bone marrow blasts, and poor-risk cytogenetics to be associated with worse survival [10]. Patients with severe thrombocytopenia had a median overall survival (OS) of 15.4 months [10], similar to that of patients with IPSS Int-2–risk disease [9]. In addition, severe anemia can lead to RBC transfusion dependence, which is associated with decreased quality of life (QOL) and increased morbidity and mortality [11, 13–16]. Currently, no approved therapies prolong survival in patients with lower-risk MDS who have both transfusion-dependent anemia and thrombocytopenia. Standard therapy for patients with lower-risk MDS with anemia remains supportive treatment with erythropoiesis-stimulating agents and RBC transfusions [17]. Erythropoiesis-stimulating agents may be beneficial for some RBC transfusion–dependent patients but have no effect on thrombocytopenia. Thrombopoietin-receptor agonists are under investigation for the treatment of thrombocytopenia in MDS; although early clinical trial results are promising, more data are needed before these agents can be recommended for routine use [18, 19]. Intravenous or subcutaneous azacitidine and intravenous decitabine are approved for treatment of lower-risk MDS in a limited number of countries and are recommended for the treatment of patients with thrombocytopenia [17]; however, few data exist regarding their use in patients with lower-risk MDS who have both transfusion-dependent anemia and thrombocytopenia [8, 20, 21]. There is a significant need for additional treatment options to improve outcomes for patients with lower-risk MDS who have poor prognosis due to the presence of multiple severe cytopenias.

Results from an alternative dosing schedule study of subcutaneous azacitidine treatment in patients with MDS, many of whom were “lower risk” according French-American-British classification, suggest that patients with multiple cytopenias may benefit from lower doses of azacitidine over a prolonged period of time [21]. Although patient numbers were small, in patients with RBC transfusion–dependent anemia, baseline thrombocytopenia did not negatively impact rates of RBC transfusion independence with a lower and more prolonged dosing schedule of azacitidine. Baseline thrombocytopenia was associated with lower rates of RBC transfusion independence in patients who received shorter dosing schedules of a higher dose of azacitidine.

It is hypothesized and preclinical data suggest that exposure to azacitidine over a prolonged period of time at lower doses may result in enhanced DNA hypomethylation [22, 23]. CC-486 is an oral formulation of the epigenetic modifier azacitidine. Oral administration allows delivery of drug over a longer schedule than can be practically achieved with an injectable agent. Therefore, extended dosing with CC-486 will eliminate the requirement of injections and improve the convenience of prolonged treatment compared with an injectable formulation. Also, extended dosing with CC-486 may prolong therapeutic effects compared with shorter dosing regimens.

A recent phase 1 trial demonstrated that CC-486 is biologically and clinically active in patients with hematologic malignancies, including MDS, chronic myelomonocytic leukemia, and AML [24–26]. Part 1 of this trial was a dose-escalation study of CC-486 administered once daily for 7 days in 28-day cycles. Azacitidine was administered subcutaneously (75 mg/m^2^/day × 7 days) in cycle 1 and then orally with CC-486 (120–600 mg/day × 7 days of every 28-day cycle) from cycle 2 forward [24]. The maximum tolerated dose of CC-486 was 480 mg for 7 days of 28-day dosing. Hematologic responses were observed in patients with MDS or chronic myelomonocytic leukemia. CC-486 also induced significant hypomethylation of gene loci in treated patients during cycle 2 vs baseline.

Part 2 of this trial examined the use of CC-486 in extended 14- or 21-day dosing schedules in 28-day treatment cycles. Part 2 was later amended to include an expanded cohort of 53 patients with IPSS lower-risk MDS with poor prognostic features: anemia or thrombocytopenia with or without RBC or platelet transfusions [25]. Treatment with CC-486 at 300 mg once daily for 14 or 21 days resulted in an overall response rate of 40 %, including hematologic improvement in 28 % of patients. Additionally, RBC transfusion independence was sustained for ≥ 56 days in 47 % of patients with baseline RBC transfusion dependence. CC-486 was relatively well tolerated, and pharmacokinetic studies showed no evidence of azacitidine accumulation. Most common grade 3/4 hematologic adverse events in the 14- and 21-day dosing schedules included neutropenia (7.7 and 18.5 %, respectively), anemia (11.5 and 7.4 %), thrombocytopenia (11.5 and 3.7 %), and febrile neutropenia (3.8 and 11.1 %). Most common grade 3/4 nonhematologic adverse events in the 14- and 21-day dosing schedules included pneumonia (15.4 and 3.7 %, respectively), diarrhea (7.7 and 11.1 %), vomiting (7.7 and 7.4 %), and cellulitis (7.7 and 18.5 %) [25]. This is consistent with the known safety profile of azacitidine for injection in patients with MDS, aside from injection-site reactions [27]. Further studies showed that extended dosing of CC-486 administered at 300 mg once daily for 14 or 21 of 28 days provide sustained DNA hypomethylation through treatment cycle end [26], especially the 21-day dosing schedule, compared with 7-day dosing schedules.

Based on results of the phase 1 trial, the current randomized, placebo-controlled phase 3 trial was initiated to investigate the efficacy and safety of CC-486 for the treatment of patients with IPSS lower-risk MDS with poor prognostic features. Eligibility criteria include RBC transfusion–dependent anemia and thrombocytopenia. This is a subgroup of lower-risk MDS with poor prognosis and no effective treatment options exist apart from transplantation. The design of the QUAZAR Lower-Risk MDS study, also known as AZA-MDS-003, is described in the next section. It will hopefully provide important clinical data to expand treatment options for patients with IPSS lower-risk MDS with poor prognosis.

## Methods/design

The AZA-MDS-003 study (Additional file 1) is a multicenter, randomized, double-blind, placebo-controlled phase 3 trial comparing efficacy and safety of CC-486 plus best supportive care with placebo plus best supportive care for patients with IPSS lower-risk MDS who have thrombocytopenia as well as RBC transfusion–dependent anemia. This is a global study currently taking place in 19 countries, with study sites in North America (United States, Canada, and Mexico), South America (Brazil), Europe (Belgium, Czech Republic, Finland, France, Germany, Italy, the Netherlands, Norway, Poland, Portugal, Spain, Sweden, and the United Kingdom), Asia (Israel), and Australia. A complete list of participating sites can be found at ClinicalTrials.gov.

### Objectives

The primary goal of the study is to evaluate RBC transfusion independence with CC-486 plus best supportive care vs placebo plus best supportive care in patients with IPSS lower-risk MDS with RBC transfusion–dependent anemia (average transfusion requirement of ≥ 2 units/28 days of RBCs) and thrombocytopenia (2 platelet counts ≤ 75 × 10^9^/L at least 21 days apart). OS with CC-486 vs placebo will be evaluated as an important secondary objective. Additional secondary objectives include platelet transfusion independence as well as duration of and time to platelet transfusion independence; RBC transfusion independence for ≥ 56 days as well as duration of and time to RBC transfusion independence; progression to AML and time to AML progression; hematologic response, including erythroid response and platelet response; clinically significant bleeding events; safety; health-related QOL (HRQOL); and healthcare resource utilization.

### Patients

Eligible patients must be aged ≥ 18 years and have a documented diagnosis of MDS according to the World Health Organization 2008 classification. Patients must have RBC transfusion–dependent anemia defined as an average transfusion requirement of ≥ 2 units/28 days of RBCs without any consecutive 28-day gaps between transfusions for a minimum of 56 days immediately preceding randomization. Transfusion data include the hemoglobin value for which the transfusion was administered and must have been ≤ 10.0 g/dL within 7 days prior to transfusion administration to be counted toward the RBC transfusion–dependent status. Patients must also have thrombocytopenia defined as 2 platelet counts that are ≤ 75 × 10^9^/L at least 21 days apart. The second confirmatory platelet count must be obtained ≤ 14 days prior to randomization. Eastern Cooperative Oncology Group performance status (ECOG PS) must be 0, 1, or 2. Patients with secondary MDS (MDS as the result of chemical injury or treatment with chemotherapy and/or radiation for other diseases) are eligible provided they fulfill all other criteria. Patients with hypoplastic MDS are allowed as long as they are not eligible for treatment with immunotherapy based on investigator’s judgment. Prior treatment with lenalidomide is allowed as long as the patient received the last dose ≥ 8 weeks prior to randomization. Patients must understand and voluntarily sign an informed consent document before any study related assessments or procedures are conducted. The investigator will obtain the informed consent and a copy of the informed consent document will be given to the patient.

Exclusion criteria include having IPSS higher-risk (Int-2– or high-risk) MDS. Patients must not have been previously treated with hypomethylating agents and must not have received prior allogeneic or autologous stem cell transplant. Patients with uncontrolled dysfunction of other organs, such as cardiac, liver, or pulmonary failure, are also ineligible.

### Study design and treatment

**Fig. 1:**
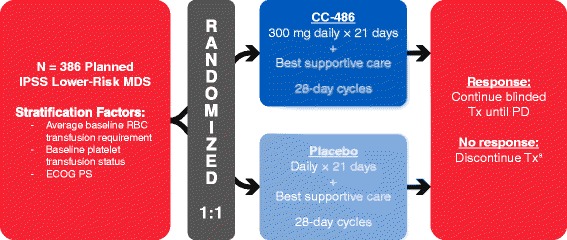
AZA-MDS-003 study design. ECOG PS, Eastern Cooperative Oncology Group performance status; IPSS, International Prognostic Scoring System; MDS, myelodysplastic syndromes; PD, progressive disease; RBC, red blood cell; Tx, treatment. ^a^ Unblinding will occur after all randomized patients have received ≥ 12 months of Tx or have discontinued from the study, whichever occurs first

This study abides by Good Clinical Practice, as described in International Conference on Harmonisation Guideline E6 and in accordance with the general ethical principles outlined in the Declaration of Helsinki. The study received approval from the institutional review boards/ethics committee of each study site prior to commencement (Additional file 2). The investigators will conduct all aspects of this study in accordance with applicable national, state, and local laws of the pertinent regulatory authorities. An estimated 386 patients will be randomized in a 1:1 ratio by a central randomization procedure using Interactive Response Technology (IRT). Investigator or designated staff will be given access to the IRT system to enroll patients. Patients will be stratified based on average baseline RBC transfusion requirement (≤4 units vs > 4 units RBC per 28 days), baseline platelet transfusion status (dependent or independent), and ECOG PS (0–1 vs 2). Patients will receive either 300 mg of CC-486 or placebo daily for the first 21 days of each 28-day treatment cycle (Fig. 1, Additional file 3). Patients, caregivers, investigators, and outcomes assessors will be blinded to treatment assignment until all randomized patients have received ≥ 12 months of treatment, or have discontinued from the study. Patients may also receive best supportive care as needed, including, but not limited to, RBC transfusions, platelet transfusions, antimicrobial therapy, nutritional support, and granulocyte colony-stimulating factors for those who experience neutropenic fever/infections and for secondary prophylaxis under certain conditions described in the protocol. Best supportive care for this study excludes the use of other antineoplastic therapies, erythropoiesis-stimulating agents, and other hematopoietic growth factors or active agents where the goal is to eradicate or slow the progression of the disease and thereby taint the ability to clearly define the efficacy endpoints.

At the end of cycle 6, disease status assessment will be performed and patients may continue treatment if they show evidence of responses including achieved RBC transfusion independence, platelet transfusion independence (for those with platelet transfusion dependence at baseline), hematologic improvement, ≥ 50 % reduction in average RBC transfusion requirements, or any other clinical benefit including no evidence of progressive disease as deemed present by the treating physician. If patients do not meet any of these criteria, they discontinue study treatment and enter the follow-up phase for safety assessment, subsequent MDS therapies, and survival. Patients may also discontinue treatment due to lack of efficacy, progressive disease, or unacceptable toxicity at any time during the study. Patients will continue to be followed until 250 deaths have occurred, after which the study will conclude.

### Efficacy assessments

The primary efficacy endpoint for this trial is the proportion of patients in the overall population achieving RBC transfusion independence with duration ≥ 84 days (12 weeks) during treatment. Baseline RBC transfusion dependency is established by RBC transfusions administered during the 56 days immediately preceding and including the date of randomization. Platelet transfusion independence with duration ≥ 56 days (8 weeks) is assessed in the proportion of patients who were platelet transfusion dependent at baseline. Baseline platelet transfusion requirements are established by the number of platelet transfusions administered during the 56 days immediately preceding and including the date of randomization. Erythroid hematologic improvement and platelet hematologic improvement will be assessed according to International Working Group 2006 criteria [28]. Hematologic response, including complete remission (CR), partial remission, marrow CR, cytogenetic response, and stable disease, will also be assessed using International Working Group 2006 criteria [28].

### Safety assessments

Safety analyses will be performed in the safety population, which includes all randomized patients who received ≥ 1 dose of any study treatment (CC-486 or placebo). Safety assessments include adverse events, monitoring for progression to AML, and second primary malignancy. Adverse events will be graded according to the Common Terminology Criteria for Adverse Events, version 4.0. Progression to AML and development of second primary malignancies will be monitored and reported as serious adverse events regardless of causal relationship to treatment with CC-486 or placebo. It is of interest to examine whether the extended exposure to CC-486 will lead to a reduction or delay in occurrence of second primary malignancies as well as progression of AML. An independent Data Monitoring Committee composed of medical oncologists/hematologists with experience in treating MDS and a statistician will evaluate safety during the course of the study.

### HRQOL assessments

The impact of CC-486 on HRQOL relative to placebo will be evaluated using the Functional Assessment of Cancer Therapy–Anemia (FACT-An) [29] and EuroQol Group EQ-5D-3 L (EQ-5D) instruments [30]. The FACT-An addresses anemia-related symptoms, including fatigue, and has been shown to discriminate between patients based on hemoglobin level and ECOG PS [29]. The EQ-5D is a non–disease-specific instrument that measures general health status [30].

### Healthcare resource utilization

Healthcare and medical resource utilization, as well as cost-effectiveness/cost-utility analyses, will be conducted. These will include reasons for and days of hospitalization, diagnostic procedures and treatment intervention not requiring hospitalization, concomitant medications, and resource use associated with treatment administration for MDS. Information will be collected through specially designed case report forms and/or routine study activities.

### Correlative assessments

Correlative assessments will be performed to determine the relationship between CC-486 exposure and endpoints, including safety, efficacy, and exploratory biomarkers. These will be assessed using population pharmacokinetic/pharmacodynamic approaches. The data collected will help validate a population pharmacokinetic model for CC-486 and aid in the identification of covariates that influence azacitidine pharmacokinetic/pharmacodynamic responses and efficacy and safety endpoints. Biomarker assessments will include DNA sequence analysis of genes identified as impacting MDS prognosis (eg, tumor protein p53 [*TP53*], enhancer of zeste homolog 2 [*EZH2*], ets variant 6 [*ETV6*], runt-related transcription factor 1 [*RUNX1*], additional sex combs like transcriptional regulator 1 [*ASXL1*], and tet methylcytosine dioxygenase 2 [*TET2*]) [31–33] and genes linked to DNA methylation (eg, *TET2*, DNA [cytosine-5-]-methyltransferase 3 α [*DNMT3A*], isocitrate dehydrogenase 1 [*IDH1*], and *IDH2*), measurement of DNA methylation levels, and immunophenotyping by flow cytometry. Bone marrow, blood, and buccal mucosa cells will be collected from patients in both control and treatment arms at screening.

### Statistical analyses

The primary efficacy analysis will be conducted in the intent-to-treat population and will compare the rates of RBC transfusion independence for ≥ 84 days with CC-486 vs placebo. RBC transfusion independence for ≥ 84 days will be analyzed and reported only once after all 386 patients have completed 12 months of double-blind treatment or have been discontinued from treatment. A stratified Mantel-Haenszel *χ*^2^ test, stratified for average baseline RBC transfusion requirement (≤4 units vs > 4 units of RBCs per 28 days), baseline platelet transfusion status (dependent or independent), country of enrollment (Japan vs rest of world), and ECOG PS (0–1 vs 2), at a 2-sided α level of 0.05, will be used to compare the RBC transfusion independence rate between the 2 treatment groups. The *P* value from the stratified Mantel-Haenszel *χ*^2^ test will be the confirmatory *P* value for the test of the null hypothesis that the proportion of patients achieving RBC transfusion independence is equal between the 2 treatment groups.

Limited information is available on the target population for estimating the response rate for the primary endpoint of RBC transfusion independence for ≥ 84 days for the active treatment group. Data from a study evaluating 3 alternative dosing regimens of subcutaneous azacitidine indicated that approximately 30 % of the subset of patients who met the criteria for the target population defined in this study achieved RBC transfusion independence for≥ 84 days while on treatment (unpublished data). For the placebo group, 2 past randomized, double-blind, placebo-controlled phase 3 trials in RBC transfusion-dependent patients with lower-risk MDS have demonstrated rates of RBC transfusion independence ≥ 56 days of 2.5 to 6 % and of RBC transfusion independence ≥ 168 to 182 days of 0 to 6 % in placebo-treated patients [34, 35]. Therefore, a total sample size of 386 patients (193 in the active treatment group and 193 in the placebo group) will have approximately 99 % power to detect the difference between a response rate of 0.30 in the active treatment group and a response rate of 0.05 in the placebo group, and approximately 90 % power to detect the difference between a response rate of 0.15 in the active treatment group and a response rate of 0.05 in the placebo group. The power calculations for response rate are based on a 2-sided α of 0.05 and test statistics on the difference of proportions using an unpooled estimate of variance.

An analysis of the OS endpoint will also be conducted at the time of the analysis of the primary efficacy endpoint of RBC transfusion independence. If the required 250 deaths have occurred (eg, study completion) at the time of the final analysis for the RBC transfusion independence endpoint, then this will be the final analysis of the OS endpoint. If not, an interim analysis of the OS endpoint will be performed and reported when all patients have completed 12 months of double-blind treatment or have been discontinued from treatment.

Power and sample-size determination will be based on a test of the equality of the OS curves between the CC-486 and placebo treatment groups using a stratified log-rank test, with the following assumptions: (1) a median OS of 18 months in the placebo-treated group, which takes into account possible crossover outside of the protocol of placebo-treated patients to active treatment during the observation period; (2) a median OS of 25.7 months (43 % improvement) in the CC-486–treated group, 386 patients (193 in each group); (3) the OS distribution is exponential with a constant failure (hazard) rate; and (4) the accrual is uniform during the accrual period (26 months) with an overall dropout rate of 1 % from both treatment groups. The study will provide approximately 80 % power to detect a constant hazard rate ratio of 0.70 using a 2-sided log-rank test with an overall significance level of 0.05. This will be achieved when approximately 250 deaths have occurred in both treatment groups, which is expected approximately 60 months after randomization of the first patient into the study. An O’Brien-Fleming group sequential-type boundary with a Lam-Demets alpha spending function will be used to preserve the overall α level of 0.05 for the analysis of OS in the event that OS is analyzed twice. The required (two-sided) significance levels are estimated to be 0.019 at the interim and 0.031 at the final analysis for OS, but the actual levels used will depend on the actual number of events at the time of the interim analysis, if conducted. The null hypothesis for testing the OS endpoint is that the OS distributions for the two treatment groups are equivalent. Overall survival curves will be estimated using Kaplan-Meier methods and will be compared using a stratified log-rank test. At the time of the final OS analysis, a stratified Cox proportional hazards model will be used to estimate the corresponding hazard ratio and 95 % CI for azacitidine relative to placebo.

Finally, with the exception of the proportion of patients progressing to AML and time to AML progression, all secondary efficacy variables remaining will be analyzed and reported once at the time of the analysis of RBC transfusion independence. Patients will continue to be followed for progression to AML and survival until 250 deaths have been observed, after which the study will conclude. Kaplan-Meier methods will be used to estimate curves for time to event secondary variables, and counts and percentages will be used to describe categorical secondary variables. Safety assessments will be summarized using descriptive statistics. An analysis of covariance will be employed to assess between-group differences in HRQOL scores, and in the proportion of patients who achieve minimal clinically important differences in HRQOL assessments. In general, missing data will not be imputed.

### Status of the trial

The first patient was enrolled in AZA-MDS-003 in May 2013, with a target enrollment of 386 patients.

## Discussion

This subgroup of patients with IPSS lower-risk MDS with both RBC transfusion–dependent anemia and thrombocytopenia has a poor prognosis with limited treatment options. Although classified as lower risk, this population has a median OS approaching that of patients with Int-2–risk disease [9, 10]. Treatment with CC-486 is being studied to assess its efficacy in improving OS, anemia, and thrombocytopenia and alleviating transfusion requirements.

Severe anemia leading to transfusion dependence has been associated with increased mortality and decreased QOL [11, 14–16]. Additionally, transfusions can lead to iron overload, which is linked to a number of morbidities and may be an adverse prognostic factor for OS and leukemia-free survival [11, 36]. Consequently, a treatment that improves or eliminates transfusion requirements may reduce transfusion-associated morbidity and mortality and improve QOL. Thus, OS and QOL are important endpoints for patients with lower-risk MDS who have multiple cytopenias and transfusion dependence. In reviewing this study, the US Food and Drug Administration strongly recommended the inclusion of OS as a secondary endpoint, and this study is statistically powered to show a significant OS difference of approximately 8 months between CC-486 treatment and placebo. In addition, HRQOL will be assessed as a secondary endpoint.

RBC transfusion dependence is associated with high healthcare resource utilization costs that may be 2 to 3 times higher than those for patients who do not receive RBC transfusions [13, 16, 37]. It is estimated that only 30 % of this difference is explained by costs directly related to transfusions [13]. Another contributing factor is that transfusion-dependent disease may be associated with higher comorbidity burden and greater disease severity. Thus, a treatment that improves or eliminates transfusion requirements may also substantially reduce associated healthcare resource utilization. This study includes an examination of medical resource utilization and cost-effectiveness/cost-utility analyses to determine the impact of CC-486 treatment on this endpoint.

Biomarkers such as DNA hypermethylation, somatic gene mutations, and aberrant immunophenotype have been linked to MDS disease pathogenesis and prognosis [31, 33, 38–43]. DNA hypermethylation is frequently observed in MDS and may lead to epigenetic silencing of tumor suppressor genes [38, 39]. Because DNA hypermethylation is blocked by azanucleosides, there is a rationale for evaluating the impact of DNA methylation levels on response to treatment with CC-486. Somatic gene mutations, including mutations in the epigenetic regulatory machinery, are also common in MDS, and point mutations have been shown to have a large prognostic impact on MDS, including IPSS lower-risk MDS [31, 33, 40]. Gene mutations will be analyzed in samples from patients enrolled in this trial to evaluate their prognostic value as well as their impact on clinical benefit of CC-486 treatment. Aberrant expression of antigens has also been demonstrated to impact prognosis of lower-risk patients with MDS [41], and a standardized flow cytometric scoring system has been developed to quantify aberrant immunophenotypes that will be used in this study [41–43].

In addition to lower-risk MDS, the CC-486 phase 3 program includes an investigation of CC-486 for the treatment of newly diagnosed AML in the maintenance setting. To date, no prior studies have shown an OS benefit in the maintenance setting. The QUAZAR AML Maintenance trial (CC-486-AML-001), a randomized, controlled phase 3 trial to evaluate CC-486 as maintenance therapy for older patients (aged ≥ 55 years) with AML who are in first CR or CR with incomplete blood count recovery following induction chemotherapy, is also ongoing (NCT01757535).

The AZA-MDS-003 study is a registration trial that will provide important clinical data that may expand treatment options with improved survival and QOL for a specific subset of poor-prognosis MDS patients: those with lower-risk MDS with anemia and thrombocytopenia. This study should also provide a better understanding of the genetic mutations that may lead to progression of disease including AML.

## Additional files

Additional file 1: Items from the World Health Organization Trial Registration Data Set. (DOCX 16 kb) Additional file 2: Ethics Committees/Review Boards Which Approved the AZA-MDS-003 Study. (DOCX 21 kb) Additional file 3: Supplementary Appendix for Protocol AZA-MDS-003, Amendment number 2.0, (08 Oct 2015). (DOCX 39 kb)

## Abbreviations

AML: acute myeloid leukemia
*ASXL1*: additional sex combs like transcriptional regulator 1
CR: complete remission
*DNMT3A*: DNA [cytosine-5-]-methyltransferase 3 α
ECOG PS: Eastern Cooperative Oncology Group performance status
*ETV6*: ets variant 6
*EZH2*: enhancer of zeste homolog 2
FACT-An: Functional Assessment of Cancer Therapy–Anemia
HRQOL: health-related quality of life
IDH: isocitrate dehydrogenase
Int: Intermediate
IPSS: International Prognostic Scoring System
MDS: myelodysplastic syndromes
OS: overall survival
QOL: quality of life
RBC: red blood cell
*RUNX1*: runt-related transcription factor 1
*TET2*: tet methylcytosine dioxygenase 2
*TP53*: tumor protein p53
