# Supplementary material for: Design and rationale of the QUAZAR Lower-Risk MDS (AZA-MDS-003) trial: a randomized phase 3 study of CC-486 (oral azacitidine) plus best supportive care vs placebo plus best supportive care in patients with IPSS lower-risk myelodysplastic syndromes and poor prognosis due to red blood cell transfusion–dependent anemia and thrombocytopenia
Source: BMC Hematol. 2016 May 3;16:12. doi: 10.1186/s12878-016-0049-5 (PMC4855808; doi:10.1186/s12878-016-0049-5)
Supplement: Additional file 1: — Items from the World Health Organization Trial Registration Data Set. (DOCX 16 kb) [file 12878_2016_49_MOESM1_ESM.docx]

**Additional File 1. Items from the World Health Organization Trial Registration Data Set**

| **Data category** | **Information** |
| --- | --- |
| Primary registry and trial identifying number | ClinicalTrials.gov NCT01566695 |
| Date of registration in primary registry | 27 March, 2012 |
| Secondary identifying numbers | AZA-MDS-003, 2012-002471-34 |
| Source(s) of monetary or material support | Celgene Corporation |
| Primary sponsor | Celgene Corporation |
| Secondary sponsor(s) | N/A |
| Contact for public queries | Associate Director, Clinical Trial Disclosure  1-888-260-1599  ClinicalTrialDisclosure@celgene.com |
| Contact for scientific queries | Associate Director, Clinical Trial Disclosure  1-888-260-1599  ClinicalTrialDisclosure@celgene.com |
| Public title | The Efficacy and Safety of Oral Azacitidine Plus Best Supportive Care Versus Placebo and Best Supportive Care in Subjects With Red Blood Cell (RBC) Transfusion-Dependent Anemia and Thrombocytopenia Due to International Prognostic Scoring System (IPSS) Low Risk Myelodysplastic Syndrome (MDS) |
| Scientific title | A Phase 3, Multicenter, Randomized, Double-Blind Study to Compare the Efficacy and Safety of Oral Azacitidine Plus Best Supportive Care Versus Placebo Plus Best Supportive Care in Subjects With Red Blood Cell Transfusion-Dependent Anemia and Thrombocytopenia Due to IPSS Lower-Risk Myelodysplastic Syndromes |
| Countries of recruitment | Australia, Belgium, Brazil, Canada, Czech Republic, Finland, France, Germany, Israel, Italy, Mexico, Netherlands, Norway, Poland, Portugal, Spain, Sweden, United Kingdom, United States |
| Health condition(s) or problem(s) studied | IPSS lower-risk myelodysplastic syndromes, red blood cell transfusion-dependent anemia, thrombocytopenia |
| Intervention(s) | Active comparator: oral azacitidine 300 mg daily (first 21 days of each 28-day cycle), best supportive care |
|  | Placebo comparator: placebo (first 21 days of each 28-day cycle), best supportive care |
| Key inclusion and exclusion criteria | Ages eligible for study: ≥ 18 years  Sexes eligible for study: both Accepts healthy volunteers: no |
|  | Inclusion criteria: adult patient (≥ 18 years); documented diagnosis of MDS; anemia that requires red blood cell transfusions; thrombocytopenia (sustained for at least 21 days) within 14 days prior to randomization; Eastern Cooperative Oncology Group (ECOG) performance status of 0, 1, or 2 |
|  | Exclusion criteria: secondary or hypoplastic MDS or other subtype with eligibility for treatment with immunotherapy; prior treatment with azacitidine, decitabine, other hypomethylating agents and lenalidomide; prior allogeneic or autologous stem cell transplant; eligible for allogenic or autologous stem cell transplant |
| Study type | Interventional |
|  | Allocation: randomized intervention model.  Assignment: Parallel  Masking: double blind (subject, caregiver, investigator, outcomes assessor) |
|  | Primary purpose: treatment |
|  | Phase 3 |
| Date of first enrollment | April 2013 |
| Target sample size | 386 |
| Recruitment status | Recruiting |
| Primary outcome(s) | Red blood cell transfusion independence (time frame: Up to 60 months; not designated as safety issue) |
| Key secondary outcomes | Number of patients alive (time frame: Up to 60 months; designated as safety issue) |
