# Supplementary material for: Design and rationale of the QUAZAR Lower-Risk MDS (AZA-MDS-003) trial: a randomized phase 3 study of CC-486 (oral azacitidine) plus best supportive care vs placebo plus best supportive care in patients with IPSS lower-risk myelodysplastic syndromes and poor prognosis due to red blood cell transfusion–dependent anemia and thrombocytopenia
Source: BMC Hematol. 2016 May 3;16:12. doi: 10.1186/s12878-016-0049-5 (PMC4855808; doi:10.1186/s12878-016-0049-5)
Supplement: Additional file 2: — Ethics Committees/Review Boards Which Approved the AZA-MDS-003 Study. (DOCX 21 kb) [file 12878_2016_49_MOESM2_ESM.docx]

**Additional File 2. Ethics Committees/Review Boards Which Approved the AZA-MDS-003 Study**

| **Study Site Ethics Committee/Review Board** |
| --- |
| Alpha Institutional Review Board |
| Alta Bates Summit Medical Center Institutional Review Board |
| AZ Sint-Jan AV - Ethisch Comité |
| Baylor College of Medicine IRB |
| Bellberry Human Research Ethics Committee |
| BRANY IRB |
| Brooke Army Medical Center lnstitutional Review Board |
| Cabrini Human Research Ethics Committee |
| Cambridge University Hospitals NHS Foundation Trust R&D |
| Cedars-Sinai Medical Center |
| CEIC Área de Salud de Salamanca |
| CEIC de Andalucia (CCEIBA) |
| CEIC de Asturias |
| CEIC de Euskadi |
| CEIC Hospital General Universitario Gregorio Marañon |
| CEIC Hospital Germans Trias i Pujol |
| CEIC Hospital La Paz |
| CEIC Hospital Santa Creu i Sant Pau |
| CEIC Hospital Universitari German Trias i Pujol |
| CEIC Hospital Universitario Ramon y Cajal |
| CEIC Hospital Vall d´Hebron |
| Central Manchester University Hospitals NHS Foundation Trust R&D |
| Centro Andaluz de Farmacovigilancia |
| Centro de Farmacovigilancia de Andalucia |
| Chesapeake Institutional Review Board |
| COFEPRIS |
| Comision de Investigacion del Hospital San Jose Tec de Monterrey y de la Division de Ciencias |
| Comissão de Ética para a Investigação Clínica - CEIC |
| Comitato di Etica della Fondazione IRCCS Ca’ Granda Ospedale Maggiore Policlinico di Milano |
| Comitato Etico Area Vasta Centro |
| Comitato Etico Az. Osp. Univ. Ospedali Riuniti Umberto I G.M. Lancisi G.Salesi |
| Comitato Etico Azienda Policlinico Umberto I |
| Comitato Etico Aziendale Azienda Ospedaliero Universitaria S. Maria Della Misericordia |
| Comitato Etico CE A.O. "Città della Salute e della Scienza" di Torino |
| Comitato Etico del Policlinico Universitario Tor Vergata |
| Comitato Etico Dell Universita Cattolica del Sacro Cuore Policlinico Universitario Agostino Gemelli |
| Comitato Etico della AUSL LE di Lecce |
| Comitato Etico dell'Azienda Ospedaliera Bianchi Melacrino Morelli |
| Comitato Etico dell'Azienda Ospedaliera Ospedali Riuniti Villa Sofia - Cervello di Palermo |
| Comitato Etico dell'Azienda Ospedaliera SS. Antonio e Biagio e Cesare Arrigo di Alessandria |
| Comitato Etico dell'Ospedale Oncologico Regionale di Rionero in Vulture |
| Comitato Etico Indipendente A.O.U. di Cagliari |
| Comitato Etico Indipendente Azienda Ospedaliera Universitaria Policlinico Tor Vergata |
| Comitato Etico Indipendente dell`Azienda Ospedaliero-Universitaria Policlinico S. Orsola-Malpighi |
| Comitato Etico Interregionale |
| Comitato Etico Milano Area B |
| Comitato Etico Palermo 2 c/o A.O.R. Villa Sofia Cervello |
| Comitato Etico per la Sperimentazione Clinica dei Medicinali A.O.U. Careggi di Firenze |
| Comitato Etico per le Sperimentazioni Cliniche dei Medicinali della Provincia di Venezia |
| Comitato Etico Regionale Unico (CERU) |
| Comitato Etico Unico Regionale - c/o Azienda Ospedaliera San Carlo |
| Comitato Etico Universita’ Cattolica S. Cuore Policlinico Gemelli |
| Comite de Etica de la Facultad de Medicina de la UANL y Hosp. Univ. Dr. Jose Eleuterio González |
| Comite de Etica em Pesquisa em Seres Humanos do Hospital Israelita Albert Einstein |
| Comitê de Ética em Pesquisa em Seres Humanos do Instituto Nacional do Câncer (INCA) |
| Comitê de Ética em Pesquisa em Seres Humanos Hospital de Clínicas de Porto Alegre |
| Comité de Etica en Investigación del Instituto Nacional de Cancerologia |
| Comite de Investigacion y Etica del Hospital Angeles de las Lomas |
| CPP Sud-Ouest et Outre-Mer I - Hôpital Purpan |
| EC CHU de Charleroi |
| Ethikkommission an der medizinischen Fakultät der Heinrich-Heine-Universität |
| Ethik-Kommission an der Medizinischen Fakultät der Universität Leipzig |
| Ethik-Kommission der Ärztekammer Hamburg |
| Ethikkommission der Ärztekammer Nordrhein |
| Ethik-Kommission der Fakultät für Medizin der Technischen Universität München |
| Ethik-Kommission der Medizinischen Fakultät "Carl Gustav Carus" der Technischen Universität Dresden |
| Ethik-Kommission der Medizinischen Fakultät der Eberhard-Karls-Universität |
| Ethik-Kommission Kiel der Medizinischen Fakultät der Christian-Albrechts-Universität zu Kiel |
| Eticka komise Fakultni nemocnice Brno |
| Eticka komise Fakultni nemocnice Hradec Kralove |
| Eticka komise Fakultni nemocnice Olomouc |
| Eticka komise Ustavu hematologie a krevni transfuze |
| Eticka komise Vseobecne fakultni nemocnice v Praze |
| Etisch Comité GZA |
| Federal Agency for Medicines and Health Products |
| Fred Hutchinson Cancer Research Center IRB |
| Gdanski Uniwersytet Medyczny Niezalezna Komisja Bioetyczna Do Spraw Badan Naukowych |
| Heart of England NHS Foundation Trust UK R&D |
| Hopital du Sacre-Coeur de Montreal Research Ethics Board |
| Hull and East Yorkshire Hospitals NHS Trust R&D |
| Human Subjects Committee - University of Kansas Medical Center |
| Institutional Helsinki Committee, Rabin Medical Center |
| Institutional Helsinki Committee, Tel-Aviv Sourasky Medical Center |
| Institutional Helsinki Committee, The Chaim Sheba Medical Center |
| Johns Hopkins Medicine Office of Human Subjects Research Institutional Review Board |
| Kaiser Permanente Northwest IRB Research Subjects Protection Office |
| Kansas City Veterans Administration Medical Center |
| Kings College Hospital NHS Foundation Trust R&D |
| Mayo Clinic IRB |
| McGill University Health Center Montreal Hospital |
| Monash Health |
| NHS Grampian UK R&D |
| Northwestern University IRB |
| Nottingham University Hospitals NHS Trust UK R&D |
| Oxford University Hospitals National Health Service Trust UK R&D |
| Phoebe Putney Memorial Hospital IRB |
| Raad van Bestuur Erasmus MC |
| Raad van Bestuur UMC St.Radboud |
| Raad van Bestuur Universitair Medisch Centrum Groningen |
| Raad van Bestuur VU Medisch Centrum |
| Rabin Medical Center Ethics Committee |
| Rabin Medical Center Local EC |
| Rambam Medical Center EC |
| Rambam Medical Center Ethics Committee |
| Rambam Medical Center Institutional Helsinki Committee |
| Regional komité for medisinsk og helsefaglig forskningsetikk, Sør-Øst-Norge (REK Sør-Øst) |
| Regionala etikprövningsnämnden i Stockholm |
| Royal Adelaide Hospital Research Ethics Committee |
| Royal Liverpool And Broadgreen University Hospitals NHS Trust UK R&D |
| Saint Luke's Hospital Institutional Review Board |
| Schulman Associates IRB |
| Schulman Associates IRB, Inc. |
| Sherwood Forest Hospitals NHS Trust UK R&D |
| Southern Health Human Research Ethics Committee A |
| Sunnybrook Health Sciences Centre Research Ethics Board |
| Tel Aviv Sourasky EC |
| The Chaim Sheba Medical Center EC |
| Therapeutic Goods Administration |
| UBC BCCA Research Ethics Board |
| University Health Network Research Ethics Board |
| University Hospital of Wales Research and Development |
| University Hospitals of Leicester NHS Trust |
| University of California, San Diego Human Research Protections Program |
| University of Chicago Hospitals Institutional Review Board |
| University of Massachusetts Medical School Committee from the Protection of Human Subjects in Research |
| University of Nebraska Medical Center Institutional Review Board |
| University of Pennsylvania Institutional Review Board |
| University of Texas MD Anderson Cancer Center Institutional Review Board |
| Urząd Rejestracji Produktów Leczniczych, Wyrobów Medycznych i Produktów Biobójczych |
| UZ Leuven - Commissie Medische Ethiek - toetsingscommissie |
| Varsinais-Suomen sairaanhoitopiirin eettinen toimikunta |
| Weill Cornell Medical College Institutional Review Board |
| Western Institutional Review Board |
