# Supplementary material for: Design and rationale of the QUAZAR Lower-Risk MDS (AZA-MDS-003) trial: a randomized phase 3 study of CC-486 (oral azacitidine) plus best supportive care vs placebo plus best supportive care in patients with IPSS lower-risk myelodysplastic syndromes and poor prognosis due to red blood cell transfusion–dependent anemia and thrombocytopenia
Source: BMC Hematol. 2016 May 3;16:12. doi: 10.1186/s12878-016-0049-5 (PMC4855808; doi:10.1186/s12878-016-0049-5)
Supplement: Additional file 3: — Supplementary Appendix for Protocol AZA-MDS-003, Amendment number 2.0, (08 Oct 2015). (DOCX 39 kb) [file 12878_2016_49_MOESM3_ESM.docx]

**Additional File 3. Supplementary Appendix for Protocol AZA-MDS-003, Amendment number 2.0, (08 Oct 2015)**

This supplementary appendix contains the following items:

Part I – ADDITIONAL METHODS: page 2

Part II – DISSEMINATION POLICY: page 6

Part III – PARTICIPANT TIMELINE: page 7

**Part I - ADDITIONAL METHODS:**

**Dose Modifications**

Patients will be monitored for hematologic and nonhematologic toxicity with the National Cancer Institute (NCI) Common Terminology Criteria for Adverse Events (CTCAE, Version 4.0) used as a guide for the grading of severity. If a certain level of toxicity is observed (eg, Table S1) and considered by the investigator to be at least possibly related to treatment, dosing of study drug may be interrupted, delayed or modified. In the event of any adverse event that would put a patient at unacceptable risk in the investigator´s opinion, dosing of study drug may be interrupted, delayed or modified even if not considered by the investigator to be at least possibly related to treatment.

**Table S1. Guidelines for Dose Modifications**

| **NCI CTCAE Toxicity Grade** | **Action** |
| --- | --- |
| Diarrhea (≥ Grade 3) | Interrupt study drug and provide adequate/maximum medical intervention  Resume study drug at same dose when toxicity resolves to ≤ Grade 1  If event reoccurs upon re-challenge or during next treatment cycle, reduce study drug dose to 200 mg.  If event reoccurs at same intensity once study drug dose is reduced to 200 mg, follow the steps above and modify the treatment schedule at a dose of 200 mg once a day. |
| Nausea and/or Vomiting  (≥ Grade 3) | Interrupt study drug and provide adequate/maximal medical intervention  Resume study drug at same dose when toxicity resolves to ≤ Grade 1  If event reoccurs upon re-challenge or at same intensity during  next treatment cycle, reduce dose to 200 mg  If event reoccurs at same intensity once study drug dose is reduced to 200 mg, follow the steps above and modify the treatment schedule at a dose of 200 mg once a day. |
| Renal Dysfunction | For unexplained elevations of serum creatinine, delay the start of the next cycle of treatment until values return to baseline. Reduce study drug dose in the next cycle of treatment to 200 mg.  The treatment schedule at a dose of 200 mg once a day can be modified if the elevation of serum creatinine recurs in the subsequent cycle.  Discontinue study drug if similar unexplained renal and/or electrolyte disturbances subsequently persist or recur during the next cycle of treatment. |

**Table S1. Guidelines for Dose Modifications (Continued)**

| **NCI CTCAE Toxicity Grade** | **Action** |  |
| --- | --- | --- |
| Febrile Neutropenia  (≥ Grade 3) | Continue study drug at the discretion of the investigator   - If episode persists for ≥ 4 days despite adequate/maximal antibiotic, antiviral and/or antifungal therapy, study drug should be temporarily interrupted. - Resume study drug at the same dose after the fever has resolved and the ANC has improved or stabilized (as assessed by the investigator). Study drug should not be resumed for at least 3 days following resolution of fever.   If a patient experiences febrile neutropenia in 2 consecutive cycles, the steps noted above should be followed, but the study drug dose should be reduced to 200 mg upon resumption of treatment. Secondary prophylaxis with G-CSF may be considered.  If a patient continues to experience febrile neutropenia episodes that are deemed to be related to the study drug by the investigator, the treatment schedule at a dose of 200 mg once a day may be modified. | |
| Neutropenia Grade 4  (related or unrelated to study drug) | **Pre-existing Neutropenia Grade 4**  Continue study drug at the discretion of the investigator   - If the neutropenia continues to worsen significantly under treatment in the investigator’s opinion, treatment may be temporarily interrupted. - Resume study drug after the ANC has improved or stabilized (as assessed by the investigator). Study drug may be reduced to 200 mg once a day at the investigator´s discretion.   If the patient continues to experience neutropenia Grade 4  during 2 consecutive cycles, the treatment schedule at a dose of  200 mg once a day may be modified. Secondary prophylaxis with G-CSF may be considered.  **Patients experiencing worsening of Neutropenia to Grade 4 under study drug treatment**  Continue study drug at the discretion of the investigator   - If episode persists for ≥ 4 days, study drug may be temporarily interrupted. - Resume study drug after the ANC has improved or stabilized (as assessed by the investigator). Study drug may be reduced to 200 mg once a day at the investigator´s discretion.   If the patient continues to experience neutropenia Grade 4 during 2 consecutive cycles, the treatment schedule at a dose of 200 mg once a day may be modified. Secondary prophylaxis with G-CSF may be considered. |  |

**Table S1. Guidelines for Dose Modifications (Continued)**

| **NCI CTCAE Toxicity Grade** | **Action** |
| --- | --- |
| Other ≥ Grade 3  non-hematologic treatment-related toxicities | Interrupt study drug dosing and provide medical intervention as appropriate  Resume study drug at same dose when toxicity resolves to ≤ Grade 2  If event reoccurs upon re-challenge or at same intensity during next treatment cycle, study drug dose may be reduced to 200 mg once a day  If event reoccurs at same intensity once study drug dose is reduced to 200 mg once a day, follow the steps above and modify the treatment schedule at a dose of 200 mg once a day. |
| ≥ Grade 2  hematologic or nonhematologic AEs putting a patient at unacceptable risk in the investigator´s opinion (related or unrelated to study drug) | Interrupt study drug dosing and provide medical intervention as appropriate  Resume study drug at same dose when toxicity resolves to ≤ Grade 1  If event reoccurs upon re-challenge or at same intensity during next treatment cycle, study drug dose may be reduced to 200 mg once a day  If event reoccurs at same intensity once study drug dose is reduced to 200 mg, follow the steps above and consider modifying the treatment schedule at a dose of 200 mg once a day. |

AE, Adverse Event; ANC, Absolute Neutrophil Count; NCI, National Cancer Institute; CTCAE, Common Terminology Criteria for Adverse Events.

**Pathology Review**

An independent central pathology reviewer will review slides of bone marrow aspirate, bone marrow biopsy and peripheral blood smear, and applicable central laboratory results prior to randomization to confirm myelodysplastic syndromes diagnosis and World Health Organization classification. If the central pathology reviewer and local pathologist disagree on the diagnosis of a patient, a third party reviewer will adjudicate and make the final assessment. For these cases the third party reviewer’s assessment will be used for the statistical analyses. The independent central pathology reviewer will also assess bone marrow aspirates, biopsies (if adequate aspirate is not attainable), peripheral blood smears and applicable central laboratory results during the study.

**Cytogenetics Review**

An independent cytogeneticist will provide standardized analysis and reporting for all patients. The independent central cytogenetic review results will be used for the statistical analyses.

**Data Management**

Data will be recorded by the investigator on the appropriate case report form (CRF) and entered into the clinical database per the sponsor’s standard operating procedures (SOPs). Programmed edit checks specified by the clinical team will be used to electronically verify the data. Any discrepancies found in the data will be brought to the attention of the clinical team, and investigational site personnel, if necessary. Resolutions to these discrepancies will be reflected in the database. All changes made to the data will be tracked within the system.

**Emergency Unblinding**

The blind must not be broken during the course of the study unless, in the opinion of the investigator, it is absolutely necessary to safely treat the patient. If it is medically necessary to know which treatment the patient is receiving, the study drug should be temporarily discontinued. The investigator may contact the Medical Monitor prior to breaking the blind to discuss unblinding, mainly in the interest of the patient. However, the decision to break the blind in emergency situations remains the responsibility of the treating physician, which will not be delayed or refused by the sponsor. The investigator should promptly notify the Medical Monitor of the emergency unblinding and the reason for breaking the blind. Emergency unblinding should only be performed by the investigator through the Interactive Response Technology (IRT) system by using an emergency unblinding personal identification number, and the investigator should access the IRT system for unblinded dose information.

**Audits and Inspections**

In addition to routine monitoring procedures, representatives from the sponsor’s Good Clinical Practice Quality Assurance unit will conduct audits of clinical research activities in accordance with the sponsor’s SOPs to evaluate compliance with Good Clinical Practice guidelines and regulations. The investigator is required to permit direct access to the facilities where the study took place, source documents, CRFs and applicable supporting records of patient participation for audits and inspections by the institutional review board (IRB)/ethics committees (ECs), regulatory authorities (eg, Food and Drug Administration [FDA], European Medicines Agency [EMA], Health Canada) and company authorized representatives.

**Confidentiality**

The sponsor affirms the patient’s right to protection against invasion of privacy and to be in compliance with the International Conference on Harmonisation and other local regulations (whichever is most stringent).

**Protocol Amendments**

Any amendment to this protocol must be approved by the sponsor’s Clinical Research Physician/Medical Monitor. Written approval from the IRB/EC for any amendment must be obtained before implementation of the amended version occurs. Amendments that are administrative in nature do not require IRB/IEC approval but will be submitted to the IRB/IEC for information purposes. In addition, if a protocol is amended and it impacts on the content of the informed consent, the informed consent document must be revised and participating patients must be reconsented with the revised version of the informed consent document.

**Adherence to Protocol-Prescribed Therapy**

Documentation of dosing during treatment will be recorded in a study specific diary card provided by the sponsor to study site personnel, who will in turn distribute them to study patients. Study site personnel will enter the scheduled daily doses, the number of tablets to be taken each day and any other applicable information. Patients (or legally authorized representative) will be asked to record study drug dosing information and anti-emetic medication taken at home in the diary card and to bring the diary card and unused tablets in the blister card (or the empty blister card packaging) with them to scheduled clinic visits. Study site personnel will perform a study drug administration compliance check and record this information in the patient’s source documentation and on the appropriate CRF.

**Part II - DISSEMINATION POLICY:**

**Publications**

The results of this study may be published in a medical publication, journal, or may be used for teaching purposes. Additionally, this study and its results may be submitted for inclusion in all appropriate health authority study registries, as well as publication on health authority study registry websites, as required by local health authority regulations. Selection of first authorship will be based on several considerations, including, but not limited to study participation, contribution to the protocol development, and analysis and input into the manuscript, related abstracts, and presentations in a study.

**Part III – Participant Timeline**

**Table S2. Study events or procedures by time point for each 28-day treatment cycle**

| **Procedure** | **Screening**  **≤ 56 Days**  **Prior to**  **Randomization** | **Random-ization** | **Double Blind Treatment Phase^a^** | | | | | | **Follow-Up^a^** |
| --- | --- | --- | --- | --- | --- | --- | --- | --- | --- |
|  |  |  | **Cycles 1-2** | | **Cycles 3-12** | | **Cycles 13 and Beyond** | **Treatment Discon-tinuation** |  |
|  |  |  | Day 1 | Days 8, 15, 22 | Day 1 | Day 15 | Day 1 |  |  |
| Informed consent | × | – | – | – | – | – | – | – | – |
| Eligibility screen | × | – | – | – | – | – | – | – | – |
| Demographics and medical history | × | – | – | – | – | – | – | – | – |
| Randomization | – | × | – | – | – | – | – | – | – |
| Oral azacitidine | – | – | Day 1 to Day 21 of 28-day treatment cycles | | | | | – | – |
| Oral placebo | – | – | Day 1 to Day 21 of 28-day treatment cycles | | | | | – | – |
| Body weight measurement | × | – | × | – | × | – | × | × | – |
| Vital signs |  |  |  |  |  |  |  |  |  |
| Physical examination  ECOG performance status | × | – | ×^b^ | – | × | – | × | × | – |
| Serum EPO level | ×^c^ | – | – | – | ×^c^ | – | – | – | – |
| Serum Ferritin^d^ | × | – | × | – | × | – | × | × | – |
| Hematology^e^ | × | – | ×^b^ | × | × | × | × | × | – |
| Serum Chemistry^e^ | × | – | ×^b^ | × | × | – | × | × | – |
| Pregnancy Testing (FCBP only)^f^ | × | – | × | – | × | – | × | × | – |
| Assessing adverse events | After signing ICD and until 28 days after the last dose of study drug or until the last study visit, whichever period is longer. | | | | | | | | |
| Monitoring for progression to AML and SPM | After signing ICD and until death, lost to follow-up, withdrawal of consent for further data collection, or study closure. | | | | | | | | |

**Table S2. Study events or procedures by time point for each 28-day treatment cycle (Continued)**

| **Procedure** | **Screening**  **≤ 56 Days**  **Prior to**  **Randomization** | **Random-ization** | **Double Blind Treatment Phase^a^** | | | | | | **Follow-Up^a^** |
| --- | --- | --- | --- | --- | --- | --- | --- | --- | --- |
|  |  |  | **Cycles 1-2** | | **Cycles 3-12** | | **Cycles 13 and Beyond** | **Treatment Discon-tinuation** |  |
|  |  |  | Day 1 | Days 8, 15, 22 | Day 1 | Day 15 | Day 1 |  |  |
| Concomitant medications, therapy, and procedures | – | From the date of randomization and until 28 days after the last dose of study drug or until the last study visit, whichever period is longer. | | | | | | | |
| Bone marrow aspirate and/or biopsy (biopsy mandatory at screening | × | – | – | – | ×^g^ | – | ×^g^ | – | – |
| Peripheral blood smear |  |  |  |  |  |  |  |  |  |
| Cytogenetic testing |  |  |  |  |  |  |  |  |  |
| Biomarker – bone marrow | ×^h^ | – | – | – | ×^g, i^ | – | ×^g, i^ | – | – |
| Biomarker – peripheral blood | ×^h^ | – |  | – | ×^g, i^ | ×^i, j^ | ×^g, i^ | – | – |
| Pharmacokinetics – peripheral blood | – | – | ×^h^ | – | ×^h, k^ | – | – | – | – |
| FACT-An and EQ-5D^l^ | – | – | × | – | × | – | × | × | – |
| Healthcare resource utilization | After signing ICD and until 28 days after the last study drug dose or until the last study visit, whichever period is longer | | | | | | | | |
| Transfusion assessment | – | – | After the date of randomization and until 28 days after the last dose of study drug or until the last study visit, whichever period is longer | | | | | | |
| Assessment of bleeding events | – | – | × | × | × | × | × | × | – |
| IWG response/improvement^m^ | – | – | – | – | ×^g^ | – | ×^g^ | – | – |
| Disease status assessment | – | – | – | – | ×^n^ | – | ×^n^ | × | – |
| Subsequent MDS therapies | – | – | – | – | – | – | – | × | ×^o^ |

**Table S2. Study events or procedures by time point for each 28-day treatment cycle (Continued)**

AML, acute myeloid leukemia; ECOG, Eastern Cooperative Oncology Group; EPO, erythropoietin; EQ-5D, EuroQol Group EQ-5D-3L; FACT-An, Functional Assessment of Cancer Therapy-Anemia; FCBP, female of childbearing potential; ICD, informed consent document; IWG, International Working Group; MDS, myelodysplastic syndromes; RBC, red blood cell; SPM, second primary malignancy.

^a^ The study visit window for visit related assessments in the double-blind treatment phase is ± 3 days for Cycles 1 and 2; ± 7 days for Cycle 3 and beyond, unless noted otherwise for a particular assessment. However, please note that in all circumstances a drug holiday of 7 days needs to be maintained for the treatment schedules of 300 mg and 200 mg for 21 days /28-day-cycle (14 and 21 days, respectively, for modified treatment schedules of 200 mg for 14 days/28-day-cycle and 7 days/28-day-cycle. Study visits should also take into account the patient’s study drug supply. Only 1 cycle of study drug will be dispensed to the patient on Day 1 of each cycle. Day 1 of Cycles 2 and beyond may be delayed from Day 28 of the prior cycle in order for patients to recover from toxicity and meet criteria for re-treatment. During follow-up, the study visit window is ± 7 days for visits scheduled monthly (including the follow-up visit 28 days after last dose if necessary) or ± 14 days for visits scheduled every 3 month. One cycle (one month) is considered as 28 days (ie, 4 weeks).

^b^ The assessment does not need to be performed if the screening examination was performed within 7 days of the first dose of study drug in the treatment phase.

^c^ Ideally, the screening serum EPO level should be collected on the same day as a planned RBC transfusion, and should not be collected within 1 week after any RBC transfusion due to possible reduction of the serum level related to the hemoglobin level achieved after the last transfusion. Serum EPO level should also be tested on Day 1 of Cycle 6. However, if possible, the sample should not be collected within 1 week after any RBC transfusion due to possible reduction of the serum level related to the hemoglobin level achieved after the last transfusion. Therefore in the event that Day 1 of Cycle 6 would be scheduled within a week of a RBC transfusion, EPO sampling may be performed on Day 1 of the following cycle.

^d^ Serum ferritin level must be collected at screening, on Day 1 of Cycle 1, on Day 1 of every 3 cycles thereafter (eg, Day 1 of Cycles 4, 7, 10, 13, etc.), and at treatment discontinuation.

^e^ The samples are to be collected at screening and prior to study drug administration, and at treatment discontinuation. Any or all laboratory assessments may be repeated more frequently if clinically indicated.

^f^ The screening pregnancy test can be used as the test prior to starting study therapy in the treatment phase if it is performed within the 72-hour timeframe. The patient may not receive study drug until the investigator has verified that the result of the pregnancy test is negative.

^g^ Day 1 of Cycles 3, 6, and 12 and every 6 months thereafter (eg, Day 1 of Cycles 18, 24, etc.).

^h^ Mandatory Biomarker sample collection. Patients must consent to the collection of these samples by signing the ICD, mandatory sample collection section.

^i^ Optional Biomarker sample collection. Samples will be collected from those patients who consent to the collection of these samples by signing the ICD, optional sample collection section.

^j^ Biomarker - Peripheral Blood collection: Day 15 (± 7 days) of Cycles 3, 6, and 12.

^k^ Two blood samples (3 mL/sample) for azacitidine PK assessment will be collected at least 2 hours apart on Day 1 of Cycles 1, 3, and 6 between 0.5 and 6.0 hours post study drug administration.

^l^ FACT-An and EQ-5D questionnaires must be completed prior to interaction with study personnel and prior to study drug administration at the start of every Cycle, and at treatment discontinuation. If blood is drawn in advance of Day 1 of a particular cycle or in advance of the treatment discontinuation visit, the questionnaires will still be completed at the first visit with the site staff at that particular cycle or treatment discontinuation visit.

^m^ IWG Response/Improvement Assessment is scheduled to be performed on Day 1 of Cycles 3, 6, and 12, and every 6 cycles thereafter (eg, Day 1 of Cycles 18, 24, etc.), and could be done at any time prior to starting the next cycle (eg, prior to starting Cycles 4, 7, 13, etc., respectively).

^n^ An assessment of disease status must be performed at the end of Cycle 6, prior to starting Cycle 7. Patients eligible for protocol-prescribed therapy beyond Cycle 6 will be assessed for disease status at the end of every cycle.

^o^ All patients discontinued from protocol-prescribed therapy for any reason should be followed for survival and subsequent MDS therapies every month for the first year following treatment discontinuation and every three months thereafter until death, lost to follow-up, withdrawal of consent for further data collection, or study closure.
